# Presence of activin signal transduction in normal ovarian cells and epithelial ovarian carcinoma

**DOI:** 10.1054/bjoc.1999.1127

**Published:** 2000-03-21

**Authors:** I Ito, T Minegishi, J Fukuda, H Shinozaki, N Auersperg, P C K Leung

**Affiliations:** Department of Obstetrics and Gynecology School of Medicine, Gunma University, Maebashi, Gunma, 371-8511, Japan; Department of Obstetrics and Gynecology, School of Medicine, Akita University, Hondo, Akita, 010-0915, Japan; Department of Obstetrics and Gynecology, University of British Columbia, Vancouver, BC, V6H 3V5, Canada

**Keywords:** activin, activin receptor, epithelial ovarian carcinoma, surface epithelial cell, Smad

## Abstract

In this study, we have investigated the expression of inhibin subunits and activin receptors (ActRs) in normal and malignant ovarian cells. Each product of the inhibin subunits (α, βa, βb) and activin receptors (ActRs) amplified by reverse transcription polymerase chain reaction were detected as a single band in human granulosa cells, surface epithelial cells (OSE), and the ovarian cancer cell lines OVCAR 3 and SKOV 3. Western blot analysis was performed using polyclonal antibodies against ActR IIa or IIb peptides based on 13 COOH-terminal amino acids; cultured human granulosa cells were used as a positive control. Using ActR IIa antibody, one major band corresponding to approximately 80 kDa and one minor band corresponding to 105 kDa were observed in the samples. One single band at approximately 60 kDa was detected in OVCAR 3 and a 50 kDa band was detected with ActR IIb antibody in cultured granulosa cell, OSE and SKOV 3. Although no detectable change was induced in Smad 4 mRNA in OVCAR 3, Smad 2 mRNA levels were increased during 48 h treatment with activin A (50 ng ml^–1^). These data provide a better understanding as the first step in the mechanism of action of the activin in the epithelial ovarian carcinoma. © 2000 Cancer Research Campaign


British Journal of Cancer (2000) 82(8), 1415–1420
© 2000 Cancer Research Campaign
DOI: 10.1054/ bjoc.1999.1127, available online at http://www.idealibrary.com on IDEAL®

### Presence of activin signal transduction in normal ovarian cells and epithelial ovarian carcinoma

**I Ito[1], T Minegishi[1], J Fukuda[2], H Shinozaki[1], N Auersperg[3] and PCK Leung[3]**

[1]Department of Obstetrics and Gynecology School of Medicine, Gunma University, Maebashi, Gunma 371-8511, Japan; [2]Department of Obstetrics and Gynecology, School of Medicine, Akita University, Hondo, Akita 010-0915, Japan; [3]Department of Obstetrics and Gynecology, University of British Columbia, Vancouver, BC V6H 3V5, Canada

**Summary** In this study, we have investigated the expression of inhibin subunits and activin receptors (ActRs) in normal and malignant ovarian cells. Each product of the inhibin subunits (α, βa, βb) and activin receptors (ActRs) amplified by reverse transcription polymerase chain reaction were detected as a single band in human granulosa cells, surface epithelial cells (OSE), and the ovarian cancer cell lines OVCAR 3 and SKOV 3. Western blot analysis was performed using polyclonal antibodies against ActR IIa or IIb peptides based on 13 COOH-terminal amino acids; cultured human granulosa cells were used as a positive control. Using ActR IIa antibody, one major band corresponding to approximately 80 kDa and one minor band corresponding to 105 kDa were observed in the samples. One single band at approximately 60 kDa was detected in OVCAR 3 and a 50 kDa band was detected with ActR IIb antibody in cultured granulosa cell, OSE and SKOV 3. Although no detectable change was induced in Smad 4 mRNA in OVCAR 3, Smad 2 mRNA levels were increased during 48 h treatment with activin A (50 ng ml$^{-1}$). These data provide a better understanding as the first step in the mechanism of action of the activin in the epithelial ovarian carcinoma. © 2000 Cancer Research Campaign

**Keywords**: activin; activin receptor; epithelial ovarian carcinoma; surface epithelial cell; Smad

Activin, inhibin, and follistatin are protein hormones, all of which were originally isolated from gonads as regulatory factors of pituitary follicle-stimulating hormone (FSH) secretion. Inhibin and activin are structurally related glycoproteins, consisting of two subunits linked by disulphide bonds (Robertson et al, 1985; Vale et al, 1986; Ying 1988; Hillier, 1991). Inhibin is composed of an α subunit and one of the two β subunits (βa or βb), whereas activin is formed from a combination of two of the same or different β subunits. The function of activin is thought to be mediated through binding to a receptor complex containing at least two proteins, referred to as type I and type II ActRs (Hino et al, 1989; Kondo et al, 1989). Cloning of cDNAs encoding the type I and II activin receptors (ActRs) in various species has shown that there are multiple isoforms for each type of receptor. In the human, two isoforms of the type I receptor, ActR Ia (Attisano et al, 1993) and ActR Ib (ten Dijke et al, 1994; Xu et al, 1994), have been identified.

Both mRNAs of inhibin subunits and ActRs have been reported to be expressed in some ovarian carcinoma cell lines, and activin has been reported to have a proliferative effect in these cells (Di Simone et al, 1996). In recent years, techniques have been developed to culture normal human ovarian epithelial cells (Kruk et al, 1990) and malignant cells from ovarian cancer patients (Hamilton et al, 1983). These studies have shown that ovarian surface epithelial cells retain sensitivity to a number of growth factors and may require their presence for proliferation. The follicular fluid released from the follicle at the time of ovulation flows over the surface of the ovary and growth factors present in the follicular fluid, including activin, may provide stimuli for the proliferation of surface epithelial cells during the healing process that follows ovulation. Thus, it is interesting to know whether OSE cells are capable of responding to activin through specific receptors.

Transforming growth factor (TGF)-β family members elicit their multifunctional effects through heteromeric complexes of type I and type II serine/threonine kinase receptors (Mathews, 1994; Massague, 1996; Derynck and Feng, 1997). Upon activin binding to a type II receptor with constitutively active kinase, the type I receptor is recruited and phosphorylated and activated by type II receptor kinase (Attisano and Wrana, 1996; Willis et al, 1996). Members of the Smad-and Mad-related (Smad) protein family are known to play pivotal roles in intracellular TGF-β family signalling (Heldin et al, 1997). Smads located in the cytoplasm are directly phosphorylated by membrane serine/-threonine kinase receptors that bind TGF-β or the related factors activin and bone-morphogenic protein (BMPs). The phosphorylated Smads then move into the nucleus as complexes that bind specific DNA sequences in target promoters, thus activating transcription.

Smad 2 and Smad 4 have been suggested as possible downstream signals of activin receptors. To determine if mRNAs for Smad 2 and Smad 4 are expressed in ovarian cancer cell lines, Northern blot hybridization was performed on the total RNA isolated from these cells. In this study, the expression of the inhibin subunits and ActRs was determined in normal and malignant ovarian cells, and the levels of Smad 2 and Smad 4 mRNA were examined in OVACAR 3 cells stimulated with activin A.

*Received 22 June 1999*
*Revised 6 October 1999*
*Accepted 2 December 1999*

*Correspondence to:* T Minegishi

### MATERIALS AND METHODS

### Cell culture and treatment

OVCAR 3 and SKOV 3, which are epithelial ovarian cancer cell lines derived from adenocarcinomas, were obtained from the American Tissue Culture Collection (ATCC, Rockville, MD, USA). Cells were cultured in medium 199 (M199) containing 10% heat inactivated fetal bovine serum (FBS) (Gibco-BRL), 100 U penicillin G $ml^{-1}$ and 100 μg streptomycin $ml^{-1}$ (Sigma Chemical Co., St Louis, MO, USA) at 37°C in 5% carbon dioxide 95% air. Then, $0.5 \times 10^6$ cells $ml^{-1}$ were plated in 60 mm culture dishes (Falcon) and cultured for 48 h. When they were near confluent, the cells were washed with M199 and medium was changed. Cells were lysed with lysis buffer (50 mM Tris–HCl pH 8.0, 150 mM sodium chloride, 0.02% sodium azide, 1% Triton X-100, 1 mg $ml^{-1}$ aprotinin, 100 mg $ml^{-1}$ phenylmethylsulphonyl fluoride) for Western blot analysis and lysed with 4 M guanidine isothiocyanate containing 1% β-mercaptoethanol (ME) for reverse transcription polymerare chain reaction (RT-PCR) analysis. To study the effects of activin on expression of Smads mRNA, OVCAR 3 cells were cultured in 60-mm dishes containing $1 \times 10^6$ cells in 5-ml M 199 with 0.1% BSA and 50 ng $ml^{-1}$ activin was added to the medium after 24 h of cell culture. Cells were further incubated and incubation was stopped at 24 h, 48 h and 72 h, and total RNA was extracted from the cultured cells.

Ovarian surface epithelium was scraped from the surface of grossly normal ovaries that were obtained with consent from patients having surgery for benign gynaecologic diseases. The cells were cultured as described previously (Kruk et al, 1990) in M 199/MDCB 105/15% FBS with 2 μl $ml^{-1}$ gentamicin and passaged with 0.06% trypsin (1:250) and 0.01% ethylenediaminetetraacetic acid when confluent. The cells' origin in ovarian surface epithelium was initially confirmed by their characteristic epithelial morphologic features and keratin expression (Siemens and Auersperg 1988). The cells when they were almost confluent in 35-mm dishes in passage 1 were lysed with lysis buffer for Western blot analysis and lysed with 4 M guanidine isothiocyanate containing 1% β-ME for RT-PCR analysis.

Human granulosa cells were collected and cultured as described previously (Li et al, 1992). Then, $0.5 \times 10^6$ cells $ml^{-1}$ were plated in 35-mm dishes and cultured for 48 h and the cells were lysed with lysis buffer for Western blot analysis and lysed with 4 M guanidine isothiocyanate containing 1% β-ME for RT-PCR analysis.

### RT-PCR and Southern blot procedures

Total RNA was isolated and extracted using the guanidinium isothiocyanate method (Chomczynski and Sacchi, 1987). The RNA concentrations were quantified by measuring the absorbance of samples at 260 nm. Complementary DNA (cDNA) was synthesized from 3 μg total RNA, using a first-strand cDNA synthesis kit (Pharmacia Ltd, Uppsala, Sweden). Synthesized cDNA (1 μl from total 15 μl) was used as template for PCR amplification where 28 cycles consisted of denaturing at 96°C for 30 s, annealing at 51°C (βa, βb subunits), 53°C (α subunit, ActR IIa), 55°C (ActR Ia), 57°C (ActR IIb) for 30 s and extension at 72°C for 1.5 min. At final cycle, a 15 min, 72°C extension step was added. The oligonucleotide primers used for amplification were derived from human inhibin α, βa, βb, ActR Ia, ActR IIa, ActR IIb cDNA sequences (Mason et al, 1986, 1989; Tanimoto et al, 1991). The sequence of each primers are previously described (Fukuda et al, 1998). Following electrophoresis in 1% agarose gels, the PCR products were visualized with an UV light by staining with 1 μg $μl^{-1}$ ethidium bromide solution. The cDNAs were transferred to nylon membranes (Hybond-N, Amersham) and fixed by UV irradiation. Blots were prehybridized for 3 h at 42°C in the presence of 50% formamide under standard conditions, and hybridized overnight at 42°C with digoxigenin-labelled cDNA probes. Inserts from the inhibin Ha4, HbA-4, GbB-5 clones (kindly supplied by Dr H Meunier) and the ActR Ia, IIa, IIb cDNA were labelled as digoxigenin with cDNA-labelling kit (Boehringer Mannheim). Complementary DNA of ActR Ia, IIa and IIb were obtained and sequenced from the PCR products from the human placenta. Under the standard protocol for the DIG luminescence detection kit (Boehringer Mannheim), the blots were exposed at room temperature to X-ray film for 2 min.

### Western blot analysis

A total of $2 \times 10^6$ OVCAR 3 cells were washed three times with ice-cold phosphate-buffered saline (PBS), and dissolved in lysis buffer by incubating for 30 min at 4°C. Following centrifuging for 5 min at 2000 ***g***, the solubilized supernatants were separated by sodium dodecyl sulphate polyacrylamide gel electrophoresis (SDS-PAGE) in the presence of 1% β-ME, using 8% gels. Proteins were transferred to Hybond C membrane (Amersham Corp.). ActR IIa, IIb were detected with polyclonal antibodies produced in rabbits by synthetic peptide from the carboxyl-terminal sequences of ActR IIa and IIb (kindly provided by Dr Vale). Bound antibodies were detected using enhanced chemiluminescent (ECL) detection kit (Amersham Corp.). The solubilized protein concentration were measured by BioRAD protein assay (BioRAD), each 25 μg protein per lane was separated by SDS-PAGE.

### Cloning of Smad 2 and Smad 4 cDNA

Oligonucleotides for PCR were designed to include all possible sequence encoding cording regions of Smad 2 and Smad 4, with the addition of *Pst* and *Echo* restriction sites for Smad 2. During PCR for Smad 2, *Pst* and *Eco*RI restriction sites were incorporated at the 5′ and 3′ ends of cDNA using primer 5′-CCACTGCAGGTTCGATACAAGAGGCTGTT-3′(1–22) and 5′-CCGGAATTCTGGATAGTAAACAGCCATAGGGACCA-3′ (1529–1554) respectively (Zhang et al, 1996).

For Smad 4, *Bam*H1 restriction site was incorporated at the 5′ end of cDNA using primer 5′-CTAGGATCCCCTTGCAACGT-TAGCTGTT-3′ (30–50) and 3′ end of cDNA 5′-AAAA-GATAAAGEAGAAGAAAAGTGA-3′ (1894–1870) respectively (Hahn et al, 1996). PCR was performed using cDNA synthesized from placental total RNA. The generated PCR products were cloned into Blueskript KS(+) and pGEM vectors. DNA sequencing were carried out using the dideoxy chain termination method.

### RNA extraction and Northern blot analysis

Total RNA was extracted from the cultured cells by the guanidium thiocyanate method (Chomczynski and Sacchi, 1987). The final RNA pellet was dissolved in dimethyl pyrocarbonate-treated

 © 2000 Cancer Research Campaign water, and total RNA was quantified by measuring the absorbance of samples at 260 nm. For Northern blot analysis, 10 μg of total RNA were separated by electrophoresis on denaturing agarose gels and subsequently transferred to a nylon membrane (Biodyne, ICN, Glen Cove, NY, USA). Northern blots were hybridized at 68°C with digoxigenin-labelled cRNA probes. In accordance with the standard protocol for the nucleic acid detection kit used (Boehringer Mannheim), membranes were then exposed to Kodak X-omat film (Eastman Kodak, Rochester, NY, USA). Smad 2 cDNA was subcloned into the Bluescript vector and linearized with *Pst*I. Smad 4 was subcloned into pGEM vector and linearized with *Sph*I. Digoxigenin-labelled cRNA probes were produced by in vitro transcription with T3 for Smad 2 or SP6 for Smad 4 RNA polymerase and with an RNA labelling kit (Boehringer Mannheim). Digoxigenin-labelled GAPDH (gluceraldehyde 3-phosphate dehydrogenase) cRNA probes were obtained by the same method. The relative abundance of a 4.0 (kb) signal for Smad 2 mRNA in different preparations were quantified with a LKB 2202 UnitroScan Laser Densitometer (LKB Producter AB, Bromma, Sweden), normalized against levels of GAPDH mRNA in each sample, and expressed as a percentage of the control value (100%). The data are presented as the mean ± s.e.m. of measurements from triplicate cultures for one representative experiment.

### Statistical analysis

Values were considered significantly different for values of $P < 0.05$ as determined by one-way analysis of variance (ANOVA).

### RESULTS

### Expression of inhibin subunit mRNAs

Each product of the inhibin subunits (α, βa, βb) amplified by RT-PCR was detected as a single band in the human granulosa cells, OSE, OVCAR 3 and SKOV 3 (Figure 1). Each size was found to correspond to the predicted fragment size. The βa subunit is only weakly expressed in OSE. The authenticity of the PCR-products was further confirmed by Southern blotting using the inhibin subunits cDNA probes.

### Expression of ActR mRNAs

ActR Ia, IIa and IIb, all amplified by RT-PCR, were detected as single bands (Figure 2). Each size was found to correspond to the predicted fragment size. The ActR Ia is minimal in granulosa cells and modest in OSE compared to the two ovarian cancer cell lines. The ActR IIb is expressed very little by all of the samples. The authenticity of the PCR-products was further confirmed by Southern blotting using the inhibin subunits cDNA probes, and ActR IIb alone was shown in Figure 2C.

### Western blot analysis of ActR IIa and IIb

Western blot analysis was performed using polyclonal antibodies against ActR IIa or IIb peptides based on 13 COOH-terminal amino acids (Figure 3). Cultured human granulosa cells were used as a positive control. Using ActR IIa antibody, one major band corresponding to approximately 80 kDa and one minor band corresponding to 105 kDa were observed in all samples except OSE. As

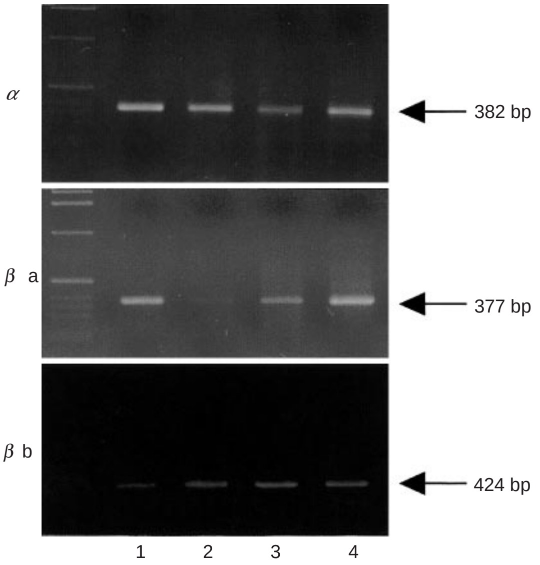

**Figure 1** PT-PCR amplification of inhibin subunits (α, β a and β b). Ethidium bromide stained PCR-product (28 cycles) were separated on 1% agarose gel. Lanes: 1, human cultured luteal-granulosa cells obtained from IVF-ET patients; 2, OSE; 3, OVCAR 3; 4, SKOV 3

shown in previous experiments (Fukuda et al, 1998), the larger molecular weight signals might be the result of an activin and ActR complex. One single band of approximately 60 kDa was detected in OVCAR 3 and a 50 kDa band was detected in the granulosa cells, OSE and SKOV 3 with ActR IIb antibody. Although the reasons for this difference have not been clarified, this effect might be due to differences in glycosylation or mutation.

### Expression of mRNAs for Smad 2 and Smad 4 in OVCAR 3 and SKOV3

Smad 2 and Smad 4 have been suggested as possible downstream signals of activin receptors. To determine if mRNAs for Smad 2 and Smad 4 are expressed in ovarian cancer cell lines, Northern blot hybridization was performed on the total RNA isolated from these cells. Two transcripts with sizes of 4.5 kb and 2.9 kb, respectively, were observed in these samples when the Smad 2 cDNA probe was used. Only one transcript size (4.2 kb) for Smad 4 was detected in both cell lines. To determine the time-course effects of activin on Smad expression, the cell lines were treated with 50 ng $ml^{-1}$ activin for various durations. No detectable changes were observed in Smad 2 and Smad 4 mRNA with activin treatment in SKOV 3 (data not shown). Although no detectable change was induced in Smad 4 mRNA during 72 h treatment with activin in OVCAR 3, Smad 2 mRNA levels gradually increased and significantly higher than that of control at 72 h (Figure 4).

### DISCUSSION

Inhibin and activins are members of the TGF-β superfamily of polypeptides that have been shown to have both growth-promoting and growth-inhibiting properties (Roberts et al, 1985; Vale et al, 1988; Bernstein et al, 1990; Berchuck et al, 1992; Fynan and Reiss, 1993). The importance of inhibin and its α-subunit in the regulation of stromal cell proliferation and tumour development has been recently demonstrated by using a homologous recombination to the α-subunit from the mouse genome (Matzuk et al,

*© 2000 Cancer Research Campaign* 

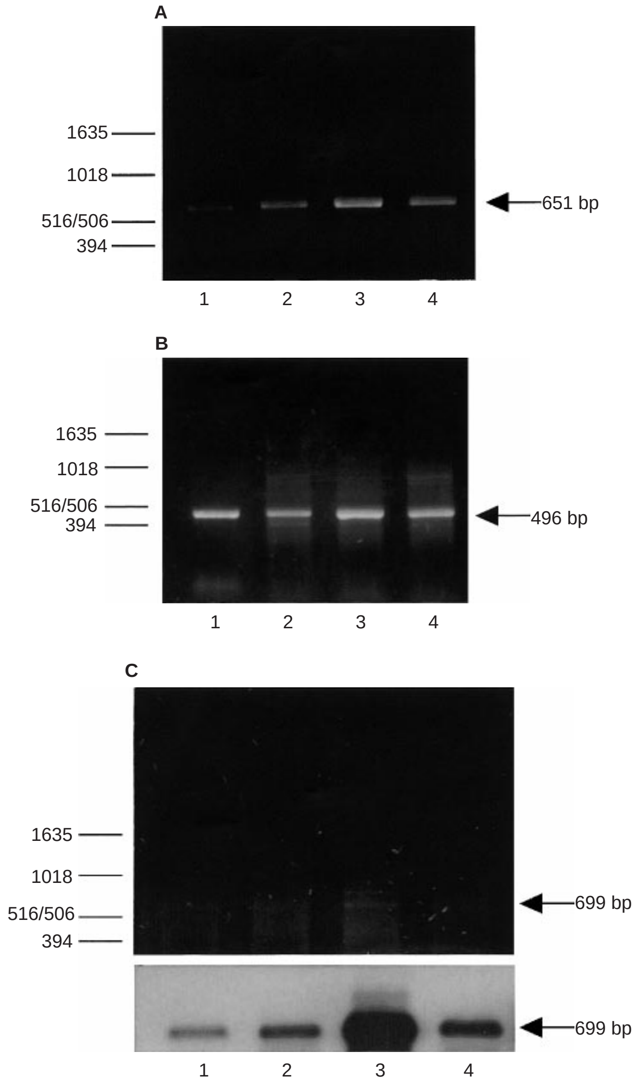

**Figure 2** PT-PCR amplification of ActRs (Ia (**A**), IIa (**B**) and IIb (**C**)). Ethidium bromide stained PCR-product for 28 cycles were separated on 1% agarose gel. Lanes: 1, human cultured luteal-granulosa cells obtained from IVF-ET patients; 2, OSE; 3, OVCAR 3; 4, SKOV 3. The PCR products were transferred to nylon membrances and subjected to Southern hybridization with ActRs (Ia (**A**), IIa (**B**) and IIb (**C**)) cDNA, and particularly ActR IIb was shown below the ethidium bromide-stained gel

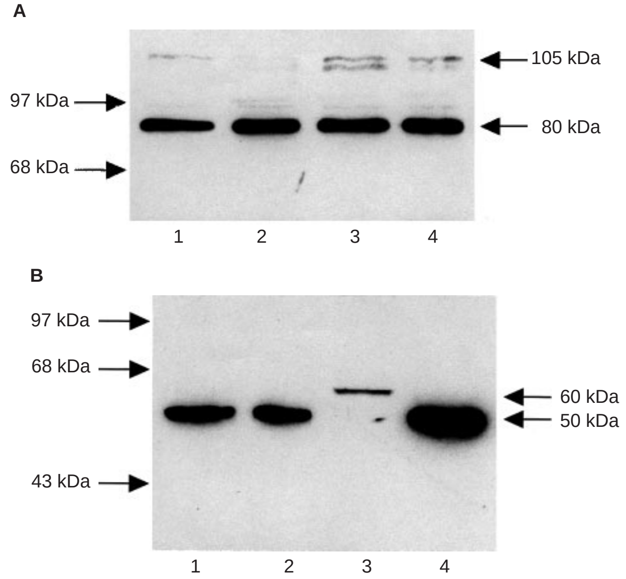

**Figure 3** Western blot analysis using ActR IIa and IIb antibodies on cultured cells. Protein was extracted from samples lysed by lysis buffer. Twenty-five micrograms protein per each lane were separated by SDS-PAGE in the presence of 1% β-ME, using 8% gels. ActR IIa (**A**) and IIb (**B**) were detected with polyclonal antibodies produced in rabbits by synthetic peptide from the carboxy 1-terminal sequences of ActR IIa and IIb. Bound antibodies were detected using an ECL detection kit. Lanes: 1, human cultured luteal-granulosa cells obtained from IVF-ET patients; 2, OSE; 3, OVCAR 3; 4, SKOV 3

1992). The loss of the inhibin α-subunit in these mice results in stromal tumour development within 6 weeks. Interestingly, the α-inhibin-deficient mice all had extremely elevated levels of circulating activin. Thus, the absence of the inhibin α-subunit or overexpression and secretion of the inhibin/activin β-subunit and dimeric activin may contribute to the development of gonadal stromal tumours.

There is a evidence suggesting that epithelial ovarian carcinomas express the activin receptors and this would indicate that activin also influences the growth of ovarian epithelial carcinomas (Welt et al, 1997). Therefore, if it were possible to clarify the presence of activin receptors in epithelial cells it might also facilitate an understanding of the mechanism of tumour development from ovarian epithelial cells. In this study, OSE cells were shown to express all of the inhibin subunits and ActR Ia, IIa and IIb mRNA, using RT-PCR and Southern blot. Furthermore, it was initially revealed that OSE expressed the ActR IIa and IIb proteins. These findings support the idea that activin and its receptors may be effective via an autocrine/paracrine mechanism acting on normal ovarian functions and on malignant growth in epithelial carcinoma. Therefore, the results of the present study support the hypothesis that potent growth factors present in the preovulatory follicle may be involved in regulating both the proliferation of the epithelial cells that cover the surface of the ovary and the subsequent development of malignant neoplasia. The healing occurring after each ovulation would require rapid mitotic activity in epithelial cells. Growth factors such as activin, which are present in the follicular fluid, may provide the stimulus for this proliferation. These same growth factors may also promote the growth of neoplastic epithelium.

Although the precise events leading to malignant transformation and progression of ovarian cancer cells are not known, it is known that multiple steps are involved. Furthermore, it has been suggested that the autonomous growth of transformed cells might be due to a constitutive expression of growth factors and membrane receptors. In this experiment, we have shown that, in addition to granulosa cells (Eramaa et al, 1995), cultured OSE cells, OVCAR 3 and SKOV 3 cells release inhibin subunits and that these cells possess receptors for activin. Improved understanding of the cellular action of receptor serine kinases has been achieved since the discovery of the newly described Smad family of proteins. Distinct subclasses of Smad proteins can be positively or negatively modulated alone or in combination with activin and TGF-β responses.

Smad 1, Smad 2, Smad 3 and Smad 5 become phosphorylated by specific, activated type I serine/threonine kinase receptors and thus these proteins act in a pathway-restricted fashion. Smad 4 forms hetero-oligomeric complexes with pathway-restricted Smad

 *© 2000 Cancer Research Campaign*

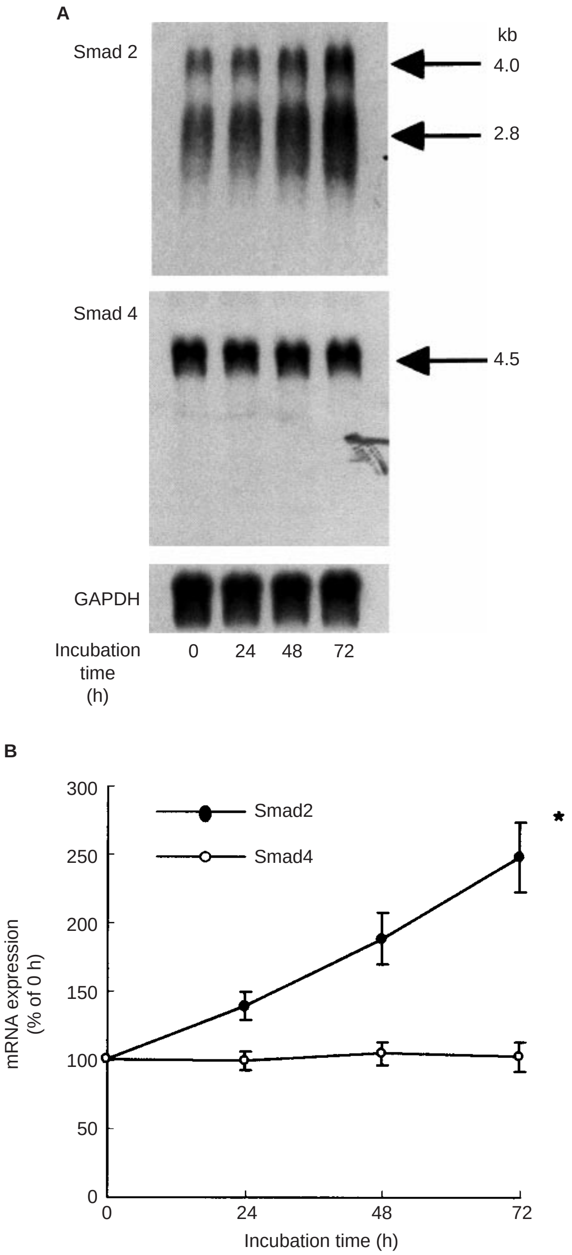

**Figure 4** Time course of activin's effect on the Smad 2 and Smad 4 mRNA in OVCAR 3. (**A**) OVCAR 3 cells were cultured for 24 h (0: control t = 0 h) in serum-free medium, and then 50 ng ml$^{-1}$ activin were added. Total RNA was extracted and Smad 2 and Smad 4 mRNA levels were measured using Northern blot analysis, as described in Materials and Methods. The Northern blot is representative of all three experiements. (**B**) Luminescence detection of Smad 2 mRNA (4.0 kb) and Smad 4 (4.5 kb) were quantified by densitometric scanning. Data were normalized for GAPDH mRNA levels in each sample and expressed relative to the control value. The absorbance values obtained from this study, as well as those from three other studies, were standardized to the control and are represented (mean ± s.e.m.; *n* = 3) in the graph. *Difference from the control value at < 0.05

proteins, which translocate into the nucleus and activate transcriptional responses. Activins act primarily through Smad 2, possibly in partnership with Smad 4, which forms heteromeric complexes with different ligand-specific Smads after activation. The principal DNA-binding component of the activin-responsive factor (ARF) is FAST-1 (Chen et al, 1996), a transcription factor with a novel winged-helix structure; previous reports indicate that Smad 4 stabilizes a ligand-stimulated Smad 2–FAST-1 complex as an active DNA-binding factor. Our data showed that OVCAR 3 expressed mRNAs of Smad 4 and Smad 2 proteins. While activin had no effect on the expression of Smad 4 in OVCAR 3, it increased the expression of Smad 2. These data suggest that activin may stimulate the production of the protein involving its own signalling system in order to enhance its own effects in OVCAR 3: an autocrine loop is clearly in operation. The enhancement of Smad 2 mRNA probably contributes to activin signal transduction in a ligand-dependent manner in OVCAR 3. A previous study demonstrated the presence of Mad 2 protein and transcript in granulosa cells and showed that its expression is up-regulated by TGF-β (Li et al, 1997). These data also suggest a possible feedback mechanism that enhances its own action. The present data support the presence of identical up-regulation mechanisms in the action of activin in the ovarian carcinoma cell line. These results permit a better understanding of the first step in the mechanism of action of the activin receptor and provide a good model for the interactions occurring in activation or inhibition of activin responses.

### ACKNOWLEDGEMENTS

This work was supported by grants from the Ministry of Education, Science, and Culture of Japan (10044235, 10877253, 10770821), Tokyo, Japan (to TM and II); and grants from the Medical Research Council (to PCKL), and the BC Health Research Foundation (to PCKL and NA).

### REFERENCES

Attisano L and Wrana JL (1996) Signal transduction by members of the transforming growth factor-beta superfamily. *Cytokine Growth Factor Rev* **7**: 327–339

Attisano L, Carcamo J, Ventura F, Weis FM, Massague J and Wrana JL (1993) Identification of human activin and TGF beta type I receptors that form heteromeric kinase complexes with type II receptors. *Cell* **75**: 671–680

Berchuck A, Rodriguez G, Olt G, Whitaker R, Boente MP, Arrick BA, Clarke Pearson DL and Bast RC Jr (1992) Regulation of growth of normal ovarian epithelial cells and ovarian cancer cell lines by transforming growth factor-beta. *Am J Obstet Gynecol* **166**: 676–684

Bernstein JR, Crowley WF Jr and Schneyer AL (1990) An improved method of purifying inhibin radioligand for radioimmunoassay. *Biol Reprod* **43**: 492–496

Chen X, Rubock MJ and Whitman M (1996) A transcriptional partner for MAD proteins in TGF-beta signalling. *Nature* **383**: 691–696

Chomczynski P and Sacchi N (1987) Single-step method of RNA isolation by acid guanidinium thiocyanate-phenol-chloroform extraction. *Anal Biochem* **162**: 156–159

Derynck R and Feng XH (1997) TGF-beta receptor signaling. *Biochim Biophys Acta* **1333**: F105–150

Di Simone N, Crowley WF Jr, Wang QF, Sluss PM and Schneyer AL (1996) Characterization of inhibin/activin subunit, follistatin, and activin type II receptors in human ovarian cancer cell lines: a potential role in autocrine growth regulation. *Endocrinology* **137**: 486–494

Eramaa M, Hilden K, Tuuri T and Ritvos O (1995) Regulation of inhibin/activin subunit messenger ribonucleic acids (mRNAs) by activin A and expression of activin receptor mRNAs in cultured human granulosa-luteal cells. *Endocrinology* **136**: 4382–4389

Fukuda J, Ito I, Tanaka T and Leung PC (1998) Cell survival effect of activin against heat shock stress on OVCAR3. *Life Sci* **63**: 2209–2220

Fynan TM and Reiss M (1993) Resistance to inhibition of cell growth by transforming growth factor-beta and its role in oncogenesis. *Crit Rev Oncog* **4**: 493–540

Hahn SA, Schutte M, Hoque AT, Moskaluk CA, da Costa LT, Rozenblum E, Weinstein CL, Fischer A, Yeo CJ, Hruban RH and Kern SE (1996) DPC4, a candidate tumor suppressor gene at human chromosome 18q21.1. *Science* **271**: 350–353

Hamilton TC, Young RC, McKoy WM, Grotzinger KR, Green JA, Chu EW, Whang Peng J, Rogan AM, Green WR and Ozols RF (1983) Characterization of a

© 2000 Cancer Research Campaign human ovarian carcinoma cell line (NIH: OVCAR-3) with androgen and estrogen receptors. *Cancer Res* **43**: 5379–5389

Heldin CH, Miyazono K and ten Dijke P (1997) TGF-β signalling from cell membrane to nucleus through SMAD proteins. *Nature* **390**: 465–471

Hillier SG (1991) Regulatory functions for inhibin and activin in human ovaries. *J Endocrinol* **131**: 171–175

Hino M, Tojo A, Miyazono K, Miura Y, Chiba S, Eto Y, Shibai H and Takaku F (1989) Characterization of cellular receptors for erythroid differentiation factor on murine erythroleukemia cells. *J Biol Chem* **264**: 10309–10314

Hurteau J, Rodriguez GC, Whitaker RS, Shah S, Mills G, Bast RC and Berchuck A (1994) Transforming growth factor-beta inhibits proliferation of human ovarian cancer cells obtained from ascites. *Cancer* **74**: 93–99

Kondo S, Hashimoto M, Etoh Y, Murata M, Shibai H and Muramatsu M (1989) Identification of the two types of specific receptor for activin/EDF expressed on Friend leukemia and embryonal carcinoma cells. *Biochem Biophys Res Commun* **161**: 1267–1272

Kruk PA, Maines Bandiera SL and Auersperg N (1990) A simplified method to culture human ovarian surface epithelium. *Lab Invest* **63**: 132–136

Li M, Li J, Hoodless PA, Tzukazaki T, Wrana JL, Attisano L and Tsang BK (1997) Mothers against decapentaplegic-related protein 2 expression in avian granulosa cells is up-regulated by transforming growth factor beta during ovarian follicular development. *Endocrinology* **138**: 3659–3665

Li W, Yuen BH and Leung PC (1992) Inhibition of progestin accumulation by activin-A in human granulosa cells. *J Clin Endocrinol Metab* **75**: 285–289

Mason AJ, Niall HD and Seeburg PH (1986) Structure of two human ovarian inhibins. *Biochem Biophys Res Commun* **135**: 957–964

Mason AJ, Berkemeier LM, Schmelzer CH and Schwall RH (1989) Activin B: precursor sequences, genomic structure and in vitro activities. *Mol Endocrinol* **3**: 1352–1358

Massague J (1996) TGFbeta signaling: receptors, transducers, and Mad proteins. *Cell* **85**: 947–950

Mathews LS (1994) Activin receptors and cellular signaling by the receptor serine kinase family. *Endocr Rev* **15**: 310–325

Matzuk MM, Finegold MJ, Su JG, Hsueh AJ and Bradley A (1992) Alpha-inhibin is a tumour-suppressor gene with gonadal specificity in mice. *Nature* **360**: 313–319

Roberts AB, Anzano MA, Wakefield LM, Roche NS, Stern DF and Sporn MB (1985) Type beta transforming growth factor: a bifunctional regulator of cellular growth. *Proc Natl Acad Sci USA* **82**: 119–123

Robertson DM, Foulds LM, Leversha L, Morgan FJ, Hearn MT, Burger HG, Wettenhall RE and de Kretser DM (1985) Isolation of inhibin from bovine follicular fluid. *Biochem Biophys Res Commun* **126**: 220–226

Siemens CH and Auersperg N (1988) Serial propagation of human ovarian surface epithelium in tissue culture. *J Cell Physiol* **134**: 347–356

Tanimoto K, Handa S, Ueno N, Murakami K and Fukamizu A (1991) Structure and sequence analysis of the human activin beta A subunit gene. *DNA Seq* **2**: 103–110

ten Dijke P, Yamashita H, Ichijo H, Franzen P, Laiho M, Miyazono K and Heldin CH (1994) Characterization of type I receptors for transforming growth factor-beta and activin. *Science* **264**: 101–104

Vale W, Rivier J, Vaughan J, McClintock R, Corrigan A, Woo W, Karr D and Spiess J (1986) Purification and characterization of an FSH releasing protein from porcine ovarian follicular fluid. *Nature* **321**: 776–779

Vale W, Rivier C, Hsueh A, Campen C, Meunier H, Bicsak T, Vaughan J, Corrigan A, Bardin W, Sawchenko P, Petraglia F, Yu J, Plotsky P, Spiess J and Rivier J (1988) Chemical and biological characterization of the inhibin family of protein hormones. *Recent Prog Horm Res* **44**: 1–34

Welt CK, Lambert Messerlian G, Zheng W, Crowley WF Jr and Schneyer AL (1997) Presence of activin, inhibin, and follistatin in epithelial ovarian carcinoma. *J Clin Endocrinol Metab* **82**: 3720–3727

Willis SA, Zimmerman CM, Li LI and Mathews LS (1996) Formation and activation by phosphorylation of activin receptor complexes. *Mol Endocrinol* **10**: 367–379

Xu J, Matsuzaki K, McKeehan K, Wang F, Kan M and McKeehan WL (1994) Genomic structure and cloned cDNAs predict that four variants in the kinase domain of serine/threonine kinase receptors arise by alternative splicing and poly(A) addition. *Proc Natl Acad Sci USA* **91**: 7957–7961

Ying SY (1988) Inhibins, activins, and follistatins: gonadal proteins modulating the secretion of follicle-stimulating hormone. *Endocr Rev* **9**: 267–293

Zhang Y, Feng X, We R and Derynck R (1996) Receptor-associated Mad homologues synergize as effectors of the TGF-beta response. *Nature* **383**: 168–172

 *© 2000 Cancer Research Campaign*
